# Meta-Analysis of Combined Therapy with Angiotensin Receptor Antagonists versus ACE Inhibitors Alone in Patients with Heart Failure

**DOI:** 10.1371/journal.pone.0009946

**Published:** 2010-04-01

**Authors:** Andrea Kuenzli, Heiner C. Bucher, Inder Anand, Gregory Arutiunov, Leo C. Kum, Robert McKelvie, Rizwan Afzal, Michel White, Alain J. Nordmann

**Affiliations:** 1 Institute for Clinical Epidemiology and Biostatistics, University Hospital Basel, Basel, Switzerland; 2 Cardiovascular Division, University of Minnesota Medical School, Veterans Affairs Medical Center, Minneapolis, Minnesota, United States of America; 3 Russian State Medical University, Moscow, Russian Federation; 4 Division of Cardiology, Prince of Wales Hospital, The Chinese University of Hong Kong, Hong Kong, Special Administrative Region, People's Republic of China; 5 Population Health Research Institute, Hamilton Health Sciences - General Site and McMaster University, Hamilton, Ontario, Canada; 6 Research Center, Montreal Heart Institute, Montréal, Quebec, Canada; 7 Université de Montréal, Montréal, Quebec, Canada; Lerner Research Institute, United States of America

## Abstract

**Background:**

There is insufficient evidence whether the benefit of adding angiotensin II receptor blockers (ARBs) to angiotensin-converting enzyme (ACE) inhibitors outweighs the increased risk of adverse effects in patients with heart failure.

**Methodology/Principal Findings:**

Two independent reviewers searched and abstracted randomized controlled trials of ARBs and ACE inhibitors compared to ACE inhibitor therapy alone in patients with heart failure reporting mortality and hospitalizations having a follow-up of at least 6 months identified by a systematic literature search. Eight trials including a total of 18,061 patients fulfilled our inclusion criteria. There was no difference between patients treated with combination therapy and ACE inhibitor therapy alone for overall mortality, hospitalization for any reason, fatal or nonfatal MI. Combination therapy was, however, associated with fewer hospital admissions for heart failure (RR 0.81, 95%CI 0.72–0.91), although there was significant heterogeneity across trials (p-value for heterogeneity = 0.04; I^2^ = 57% [95%CI 0–83%]). Patients treated with combination therapy had a higher risk of worsening renal function and symptomatic hypotension, and their trial medications were more often permanently discontinued. Lack of individual patient data precluded the analysis of time-to-event data and identification of subgroups which potentially benefit more from combination therapy such as younger patients with preserved renal function and thus at lower risk to experience worsening renal function or hyperkalemia.

**Conclusions/Significance:**

Combination therapy with ARBs and ACE inhibitors reduces admissions for heart failure in patients with congestive heart failure when compared to ACE inhibitor therapy alone, but does not reduce overall mortality or all-cause hospitalization and is associated with more adverse events. Thus, based on current evidence, combination therapy with ARBs and ACE inhibitors may be reserved for patients who remain symptomatic on therapy with ACE inhibitors under strict monitoring for any signs of worsening renal function and/or symptomatic hypotension.

## Introduction

Congestive heart failure is a major and growing public health problem in the United States. Approximately 5 million patients suffer from congestive heart failure, and over half a million patients are newly diagnosed with congestive heart failure each year [1]. The disorder is the primary reason for 12 to 15 million office visits and 6.5 million hospital days each year [1]. The estimated direct and indirect cost of congestive heart failure in the United States for 2006 was $29.6 billion [2].

Several therapeutic approaches in congestive heart failure management have led to an important reduction of cardiovascular morbidity and mortality like the blockade of the renin-angiotensin system by angiotensin-converting enzyme (ACE) inhibitors [3]–[7]. However, ACE inhibitors are unable to completely block the persistent activation of the renin-angiotensin system [8], [9] due to the existence of ACE-independent pathways (e.g., chymase, cathepsin, and kallikrein) converting angiotensin I to angiotensin II.

Therefore, the combination of ACE inhibitors and angiotensin II receptor blockers (ARBs) has been propagated for more complete blockade of the renin-angiotensin system [10], [11]. The combination of ACE inhibitors and ARBs decreases more effectively the plasma concentrations of aldosterone and brain natriuretic peptide than either ACE inhibitors or ARB alone [12], [13]. The addition of ARB to background therapy with ACE inhibitors has an additional attenuating effect on LV remodeling [14], and thus offers the potential to reduce cardiovascular morbidity and mortality in patients with congestive heart failure.

However, combining ACE inhibitors and ARBs may cause important adverse effects. In 2 recently published meta-analyses the combination of ARBs and ACE inhibitors was associated with more adverse effects as compared to ACE inhibitor therapy alone [15], [16]. However, both meta-analyses focussed on adverse effects associated with combination therapy and did not address outcomes such as readmission for heart failure or mortality where combination therapy may offer a benefit over ACE inhibitor therapy alone. One earlier published meta-analysis indicated a benefit from combination therapy compared to ACE-inhibitor alone on readmission rates for heart failure [17], but failed to report overall readmission rates which are of particular interest based on the observed increase in adverse effects observed in the 2 meta-analyses mentioned above. Another meta-analysis limited its analysis to overall mortality and a combined outcome of overall mortality and morbidity [18]. There was no difference in overall mortality. For some reasons, authors did not provide information about which individual outcomes they summarized under the term “morbidity”. Thus, in patients with congestive heart failure it remains unclear whether any potential benefit of combination therapy on outcomes may be outweighed by an increase in adverse events. In order to resolve this issue, we conducted a comprehensive meta-analysis to investigate the effect of adding ARBs to ACE inhibitor therapy alone in terms of clinically relevant beneficial and adverse patient important outcomes including hospital readmissions for any reason.

## Methods

Eligibility criteria for this meta-analysis were randomized controlled trials comparing combined ARB and ACE inhibitor therapy to ACE inhibitor therapy alone in patients with left ventricular dysfunction or congestive heart failure, with a minimal 6 months follow-up that reported mortality and hospitalization outcomes. For eligible trials we required a background therapy with ACE inhibitor therapy in at least 90% of patients.

### Data sources and search

The electronic databases MEDLINE, EMBASE, PASCAL (all from their inception to December 2009) and the Cochrane Central Register of Controlled Trials were searched for the terms “Angiotensin-Converting Enzyme Inhibitors”, “Angiotensin II Receptor Blockers” as text words and “Angiotensin-Converting Enzyme Inhibitors”, “Angiotensin II Type 1 Receptor Blockers”, “losartan”, “valsartan”, “candesartan”, “irbesartan”, “eprosartan”, “olmesartan”, “telmisartan”, and “receptors, angiotensin/antagonists and inhibitors” as Medical Subject Headings. We restricted the search to articles indexed as clinical trial (publication type) or those that included the words *random* or *placebo* in their titles or abstracts. No language restrictions were imposed. We also searched reference lists of identified articles, clinical trial register of ongoing or planned trials, recently published editorials and reviews on the topic for further eligible trials. Authors of included primary trials were asked to contribute additional data relevant for the purpose of this analysis.

### Selection and quality assessment

Two authors independently assessed trial eligibility and quality. We assessed the quality of trials according to concealment of treatment allocation, blinding of patients, caregivers, or clinical outcome assessors, full description of losses to follow-up and withdrawals and the proportion of patients with complete clinical follow-up [19]. We considered treatment allocation to be concealed if a central independent randomization facility, the use of numbered sealed opaque envelopes, or a central pharmacy which prepared and distributed numbered containers were mentioned in the report.

### Endpoints and data extraction

Two authors (AK, AN) independently extracted in duplicate all trial data and the additional data provided by the original investigators. Endpoints and adverse effects were considered irrespective of their putative relation to the treatment. We assessed the following clinical endpoints at the latest time of follow-up available: Total mortality, hospitalizations for heart failure (defined as number of distinct patients with rehospitalization for heart failure), hospitalizations for any reason (defined as number of distinct patients with rehospitalization for any reason), nonfatal myocardial infarction (MI), fatal MI, revascularisation procedures (PCI, CABG), fatal and non-fatal strokes, and quality of life.

We also gathered information about the frequency of the following adverse effects as defined by individual trials' definitions: Worsening renal function, symptomatic hypotension, hyperkalemia, cough, rash, angioedema and permanent discontinuation of study medication.

### Statistical analysis

We pooled treatment effects and calculated risk ratios for all clinical endpoints in the treatment and control groups by using a random effects model [20]. The presence of publication bias was investigated by means of funnel plots [21]. We tested for heterogeneity with the Cochrane Q test and measured inconsistency (I^2^; the percentage of total variance across studies that is due to heterogeneity rather than chance) of treatment effects across all clinical endpoints and averse effects [22], [23]. We conducted sensitivity analyses to examine treatment effects according to: quality components of included trials (concealed treatment allocation, blinding of patients and caregivers, blinded outcome assessment); trials including patients with acute MI versus trials including patients without acute MI; trials with a clear specification to achieve target doses and where ≥80% of patients in the combination group reached these target dose of the prescribed ARB versus trials where < than 80% of patients reached the target dose of the prescribed ARB; trials where ≥50% versus trials where <50% of included patients received beta-adrenergic antagonists; limitation of analysis to trials including more than 100 patients, and trials including patients with different causes (ischemic versus non-ischemic) and severity of congestive heart failure (NYHA I and II versus NYHA III and IV). We used Stata 9.2 (StataCorp, College Station/Texas) for data analysis.

## Results

**Figure 1:**
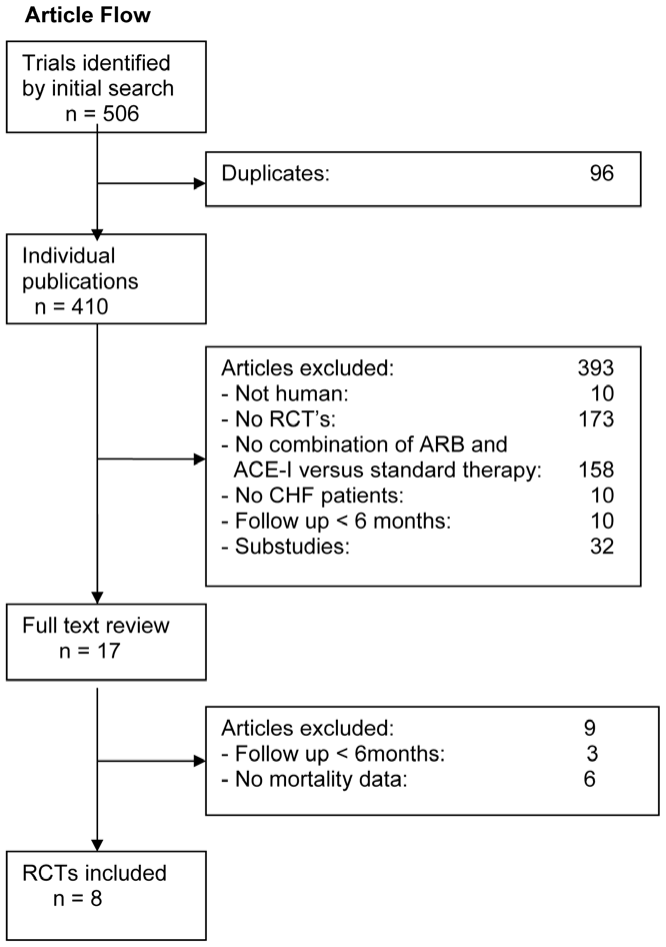
Selection process of included trials. ACE-I angiotensin-converting enzyme inhibitor, ARB angiotensin II receptor antagonist, RCT randomized controlled trial.

Eight trials including a total of 18 061 patients fulfilled our inclusion criteria [Figure 1 - selection process of included trials]. The relatively small number of trials precluded a sensitive exploration of publication bias, although the plot of standardized effect against precision did not indicate evidence for such a bias (p>0.8) [21]. Characteristics of included trials are summarized in Table 1.

**Table 1:** Trial characteristics of randomized controlled trials comparing combination therapy with angiotensin II receptor antagonists and ACE inhibitor therapy versus ACE inhibitor therapy alone in patients with congestive heart failure.

Trial	Year	Study size (n)	Inclusion criteria	Primary and secondary endpoints	Recommended target dose of ARB in combination therapy (% of patients at target dose)	Recommended target dose of ACE inhibitor therapy	Follow-up period	Severity of heart failure (combination/ACE inhibitor therapy alone, %)
Hamroff et al.	1999	33	Treatment with digoxin, diuretics, and ACE inhibitor at maximally recom-mended or tolerated dosis for ≥3 months	Peak oxygen up-take, NHYA functional class	Losartan 50mg once daily (100%)	Captopril 50mg three times daily	0.5 yr	NYHA III and IV
RESOLVD	1999	441	EF <40%, 6 minutes walking distance <500m	Six minutes walking distance, ventricular volumes, neurohormone levels, quality of life, NYHA	Candesartan 16mg once daily (>80%)	Enalapril 10mg twice daily	0.8 yr	NYHA II and III (I patient with NYHA IV)
Arutiunov GP et al.	2000	105	Acute myocardial infarction with symptomatic heart failure in hemo-dynamically stable patients, EF ≤40%, stable, and optimal dose of ACE-I plus β-blocker for at least 3 months before enrolment	Mortality, changes in severity of heart failure	Not indicated	Not indicated	2.5 yrs	NYHA II and III
Val-HeFT	2001	5010	EF <40%, clinically stable on fixed dose regimen for at least 2 weeks that could include ACE-I, diuretics, digoxin, β-blockers	Mortality/com-bined endpoint of mortality and morbidity (cardiac arrest with resuscitation, heart failure hospitalisation, or administration of intravenous ino-tropic or vasodilator drugs for ≥4 hours without hospitalisation)	Valsartan 160mg twice daily (84%)	Not indicated	2 yrs	NYHA II and III (4 patients with NYHA IV)
CHARM Added	2003	2548	EF ≤40%, treatment with an ACE inhibitor ≥30 days at constant dose	Composite of cardiovascular death or hospital admission for CHF	Candesartan 32mg once daily (61%)	No recommended fixed dose, but investiga-tors were advised of the doses of ACE inhibitors known to redu-ce morbidity and mortality in patients with congestive heart failure	3.4 yrs	Mostly NYHA II and III (7 patients with NYHA IV)
VALIANT,	2003	9794	Myocardial infarction 0.5 to 10 days previously, complicated by clinical or radiologic signs of heart failure, EF ≤35%, systolic BP >100mmHg and serum creatinine <221µmol/l	Death from any cause	Valsartan 80mg twice daily (47%)	Captopril 50mg three times daily	2 yrs	NA
White M et al.	2007	80	Symptomatic heart failure, EF <40%, optimal and stable dose of ACE-inhibitors and β-blockers for at least 3 months	Effect on N terminal protype natriuretic peptide and selected markers of inflammation and oxidative stress	Candesartan 32mg (97%)	Not indicated	0.5 yr	NYHA II and III (2 patients with NYHA IV)
Kum L et. al.	2008	50	Chronic stable heart failure, EF <50%, on regular ACE inhibitor therapy for at least 3 months	Clinical and echocardio-graphic assessment	Irbesartan 300mg (% at target dose not indicated)	Not indicated	1 yr	NYHA II and III

Abbreviations: ARB angiotensin II receptor antagonist, ACE angiotensin-converting enzyme, BP blood pressure, CHF congestive heart failure, db double blinded, EF ejection fraction, f/u follow up, LVEF left ventricular ejection fraction, N no, NA not available, NYHA New York Heart Association, sb single blinded, Y yes, *open intervention, staff blinded.

Follow-up periods of individual trials ranged from 6 to 41 months. Mean age of enrolled patients ranged from 54 to 69 years. The majority of the included patients were men (range from 48 to 94%). The vast majority of included patients had congestive heart failure NYHA class II–III with mean left ventricular ejection fraction between 25% and 35%. Most included patients (82%) had ischemic heart failure, followed by idiopathic (13%) and hypertensive heart failure (3%). Diabetes was present in 25%, hypertension in 40% of patients. Baseline characteristics of included patients are summarized in Table 2.

**Table 2:** Baseline characteristics of patients enrolled in randomized controlled trials comparing combination therapy with angiotensin II receptor antagonists and ACE inhibitor therapy versus ACE inhibitor therapy alone in patients with congestive heart failure.

Trial	Intervention	Total n	Males %	Age ± SD	Diabetes %	Hypertension %	Smoking %	Prior MI %	Ischemic heart disease %	EF %
Hamroff et al.	Combination therapy ACE-I alone	16	3165	62±13	3147	5676	2529	--	3129	27±226±2
RESOLVD	Combination therapyACE-I alone	332109	8590	64±1163±12	2432	4042	428	6773	7074	28±1127± 9
Arutiunov GP et al.	Combination therapyACE-I alone	3579	6067	68±662±7	1121	6059	4970	8669	1431	3332
Val-HeFT	Combination therapyACE-I alone	25112499	8080	62±1163±11	2625	--	--	--	5857	27±728±7
CHARM Added	Combination therapyACE-I alone	12761272	7979	64±1164±11	3030	4849	1519	5655	6263	28±828±8
VALIANT	Combination therapyACE-I alone	48854909	69.568.7	65±1265±12	2423	5555	3232	2827	100100	35±1035±10
White M et al.	Combination therapyACE-I alone	4139	9387	63±963±8	3923	3436	--	--	8877	26±728±7
Kum L et. al.	Combination therapyACE-I alone	2525	7668	66±1169±10	3240	2016	--	3644	6864	30±1234±13

ACE-I angiotensin-converting enzyme inhibitor, EF ejection fraction, MI myocardial infarction, n number, SD standard deviation, NYHA New York Heart Association.

There were two post-myocardial infarction (MI) trials. In VALIANT [24] patients were included if the ejection fraction was ≤35% within 12 hours to ten days after an acute MI and accompanied by clinical or radiological signs of congestive heart failure. Another trial [25] included hemodynamically stable patients with an ejection fraction ≤35% from 72 to 96 hours after an acute MI.

In the RESOLVD trial [26], patients were first randomly assigned to enalapril, candesartan or their combination, and in a second step after five months then randomly allocated to receive metoprolol or placebo in addition.

Three trials required patients to be on ACE inhibitor therapy for at least one [27] or three months [28], [29]. Two trials used a two to four week run-in-phase [26], [30].

Only four trials aimed at reaching the maximum recommended dose of the ARB [27]–[30] [Table 1]. The percentage of patients reaching individual trials' target dose of ARBs ranged from 47% [27] to 100% [31]. As for standard therapy, four trials aimed at reaching maximum recommended target dose of individual ACE inhibitor therapy [24], [26], [28], [31]. The percentage of patients reaching the maximum recommended dose of ACE inhibitor therapy ranged from 53% [31] to 94% [28]. In contrast, the other four trials [25], [27], [28], [30] did not report recommended fixed target doses of ACE inhibitor therapy, although in one trial clinicians were advised to target the doses of ACE inhibitors known to reduce morbidity and mortality in patients with congestive heart failure [27]. The type and dose of ACE inhibitor therapy used in either treatment groups were comparable in all but one trial [31], where the average dose of the ACE inhibitors used was lower in the standard therapy group. In one trial dosage of ACE inhibitors was not reported [29].

Concomitant therapy varied among the different trials [Table 3]. With the exception of one trial [25], at least half of the patients were taking diuretics and digoxin. The use of beta-adrenergic antagonists in individual trials varied widely from 6% [31] up to 95% [28] of patients. Only one trial [29] reported on the use of implantable cardiac defibrillators at the time of study enrolment (4% of included patients). Outcomes of individual trials are summarized in Table 4.

**Table 3:** Co-medication in randomized controlled trials comparing combination therapy with angiotensin II receptor antagonists and angiotensin-converting enzyme inhibitor therapy versus ACE inhibitor therapy alone in patients with congestive heart failure.

Trial, year	Intervention	β-Blocker %	Spironolactone %	Digoxin %	Aspirin %	Warfarin/ Marcoumar %	Lipid-lowering drug %	Calcium Antagonist %	Diuretic %
Hamroff et al., 1999	Combination therapy	6	NA	94	38	19	NA	6	100
	ACE-I alone	6	NA	100	29	35	NA	6	100
RESOLVD,1999	Combination therapy	13	NA	64	56	32	NA	15	84%
	ACE-I alone	23	NA	79	47	30	NA	14	87
Arutiunov GP et al., 2000	Combination therapy	11	NA	NA	20	NA	NA	0	9
	ACE-I alone	17	NA	NA	20	NA	NA	0	30
Val-HeFT, 2001	Combination therapy	44	NA	67	NA	NA	NA	NA	86
	ACE-I alone	36	NA	68	NA	NA	NA	NA	85
CHARM Added, 2003	Combination therapy	55	17	58	51	38	41	10	90
	ACE-I alone	56	17	59	52	38	41	11	90
VALIANT, 2003	Combination therapy	70	9		91	NA	34	NA	50
	ACE-I alone	70	9		91	NA	34	NA	49
White M, et al., 2007	Combination therapy	95	40	61	NA	NA	68	7	80
	ACE-I alone	92	44	64	NA	NA	87	8	82
Kum L et al., 2008	Combination therapy	64	0	12	68	0	60	NA	88
	ACE-I alone	64	0	8	64	0	48	NA	84

ACE-I angiotensin-converting enzyme inhibitor, NA not available.

**Table 4:** Number of events in randomized controlled trials comparing combination therapy with angiotensin II receptor antagonists and angiotensin-converting enzyme inhibitor therapy versus ACE inhibitor alone in patients with congestive heart failure.

Study	Year	Follow-up period (years)	Treatment	Total (n)	Death (n)	Non-fatal MI (n)	Fatal MI (n)	Patients with hospital admis-sion for CHF (n)	Patients with hospital admis-sion for any reason (n)	Worsening renal function (n)	Symptomatic hypotension (n)	Hyper-kalemia (n)	Permanent discontinua-tion of study medication (n)
Hamroff et al.	1999	0.5	Combination	16	0	NA	NA	NA	NA	0	0	0	1
			ACE-I alone	17	1	NA	NA	NA	NA	0	0	0	1
RESOLVD	1999	0.8	Combination	332	28	4	2	31	80	32	64	29	25
			ACE-I alone	109	5	0	0	7	24	6	13	4	6
Arutiunov GP et al.	2000	2	Combination	35	8	9	1	15	NA	1	0	1	1
			ACE-I alone	70	25	15	3	57	NA	1	0	7	3
Val-HeFT	2001	2	Combination	2511	495	77	26	346	923	28	33	N/A	249
			ACE-I alone	2499	484	75	28	455	1189	5	20	N/A	181
CHARM Added	2003	3.4	Combination	1276	377	44	18	323	852	100	58	44	309
			ACE-I alone	1272	412	69	21	382	858	52	40	9	233
VALIANT	2003	2	Combination	4862	941	677	79	834	2622	293	974	69	1139
			ACE-I alone	4879	958	720	78	945	2709	188	623	47	1055
White M et al.	2007	0.5	Combination	41	0	0	0	NA	NA	1	2	NA	4
			ACE-I alone	39	1	1	1	NA	NA	0	0	NA	4
Kum L et. al.	2008	1	Combination	25	2	0	1	4	12	0	0	0	NA
			ACE-I alone	25	1	1	1	7	11	0	0	0	NA

ACE-I angiotensin-converting enzyme inhibitor, MI myocardial infarction, n number, NA not available.

### Quality of the trials

Five trials reported concealed treatment allocation [24], [25], [27], [29], [30]. All but two trials [25], [29] used a double blind design. Blinded outcome assessment was reported in three trials [24], [27], [29] [Table 1]. Full description of losses to follow-up and withdrawals was reported in all but 2 trials [28], [30]. All trials had a loss to follow-up <10%. The 2 reviewers were in full agreement when rating the quality criteria assessed.

### Overall Mortality

**Figure 2:**
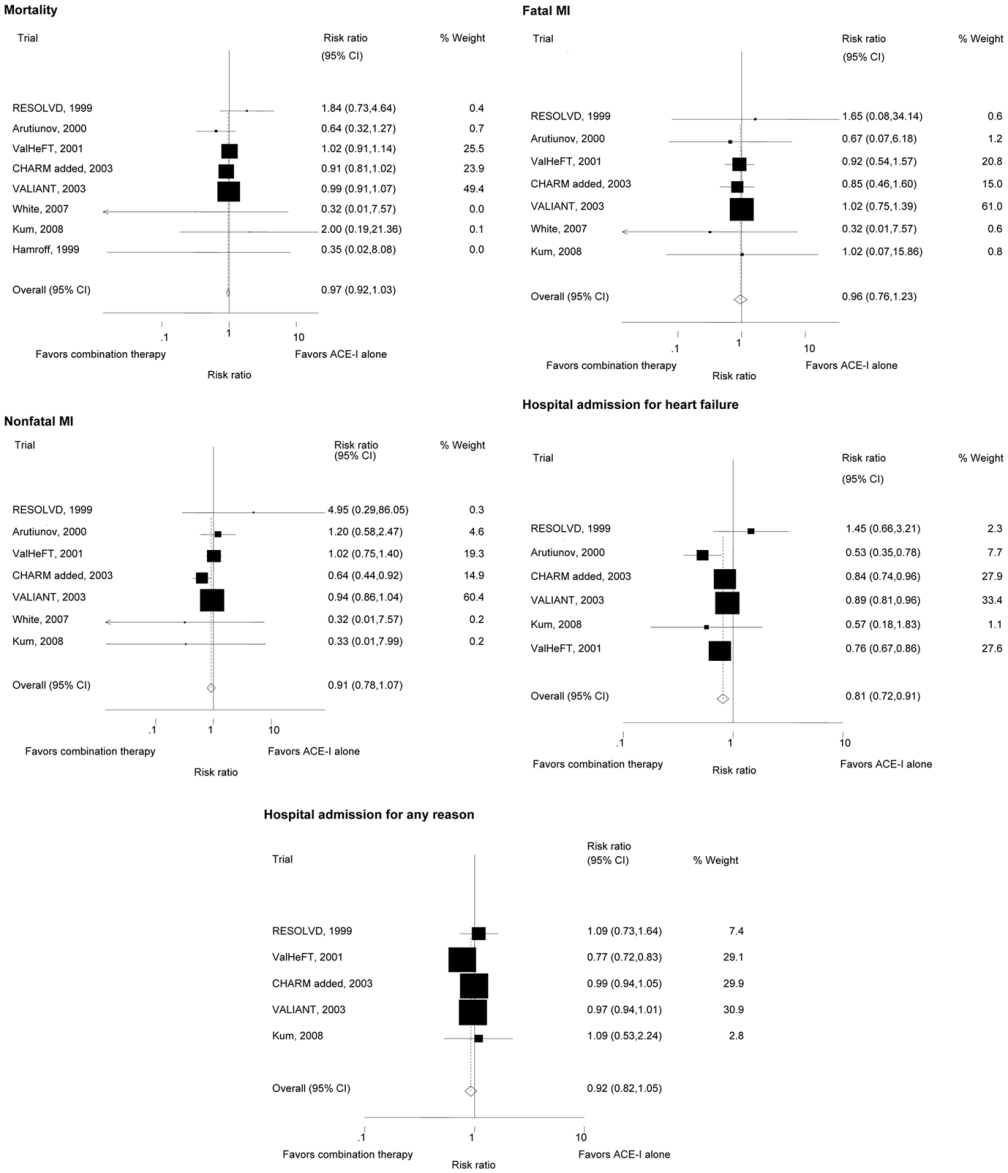
Mortality and cardiovascular outcomes in randomized controlled trials comparing angiotensin receptor antagonists and ACE inhibitors versus ACE inhibitors alone in patients with heart failure.

There was no difference in overall mortality between patients treated with combination therapy compared to ACE inhibitor therapy alone (RR 0.97, 95%CI 0.92–1.03, p-value for heterogeneity = 0.49; I^2^ = 0% [95%CI 0–68%]) [Figure 2 - mortality and cardiovascular outcomes in randomized controlled trials comparing angiotensin receptor antagonists and ACE inhibitors versus ACE inhibitors alone in patients with heart failure].

### Cardiovascular Endpoints and Quality of Life

Data on hospital admission for congestive heart failure were available from all but 2 small trials [28], [31]. There were fewer patients with combination therapy compared to ACE inhibitor therapy with hospital admissions due to congestive heart failure (RR 0.81, 95%CI 0.72–0.91; p-value for heterogeneity = 0.04; I^2^ = 57% [95%CI 0–83%]), but there was no difference between groups for hospitalization for any reason (RR 0.92, 95% CI 0.82–1.05; p-value for heterogeneity <0.001, I^2^ = 91% [95%CI 81–95%]).

There was no difference between patients treated with combination therapy and ACE inhibitor therapy alone for the relative risks of fatal (RR 0.97, 95%CI 0.76–1.22, p-value for heterogeneity = 0.97; I^2^ = 0% [95%CI 0–71%] and non-fatal MI (RR 0.91, 95%CI 0.78–1.07, p-value for heterogeneity = 0.31; I^2^ = 0% [95%CI 0–60%]). There was insufficient data for the endpoints revascularization procedures and strokes.

Five trials [24], [26], [27], [29], [30] reported quality of life data. Due to different quality of life scores used, it was not possible to pool the results. Two trials found a significant difference in favour of the combination group using the Minnesota Living with Heart Failure Questionnaire [29], [30]. In the other three trials [24], [32], [33] there was no difference between the quality of life of patients randomized to combination or ACE inhibitor therapy alone. One of these trials used the McMaster overall treatment evaluation [32], the other the EuroQol-5D preference and visual analogue scale score to measure quality of life [33]. One trial did not report how quality of life was measured [26].

### Adverse Effects

**Figure 3:**
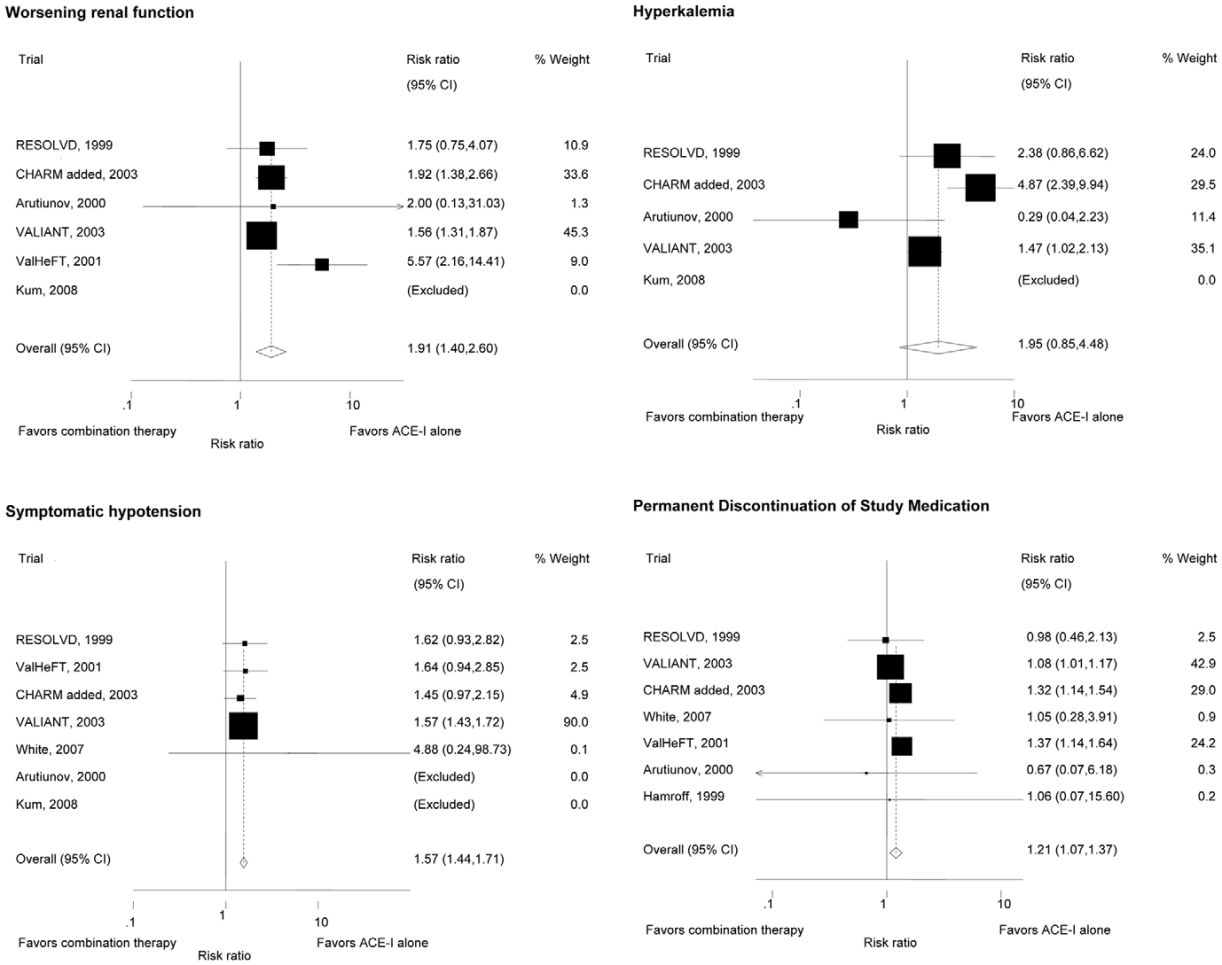
Adverse effects in randomized controlled trials comparing angiotensin receptor antagonists and ACE inhibitors versus ACE inhibitors alone in patients with heart failure.

The reporting on adverse effects was inconsistent between trials and details are provided in Figure 3 [adverse effects in randomized controlled trials comparing angiotensin receptor antagonists and ACE inhibitors versus ACE inhibitors alone in patients with heart failure]. Two trials reported on the worsening of renal function defined as a ≥50% increase of serum creatinine from baseline [26], [27]. One trial [24] defined worsening renal failure as one of the following: death from renal failure, end-stage renal disease requiring chronic dialysis or renal transplantation, or an increase in serum creatinine concentration leading to temporary or permanent discontinuation of study medication. In all other trials worsening of renal function was not defined. No trial provided an exact definition of symptomatic hypotension. Hyperkalemia was defined as a serum potassium level ≥5.5mmol/l in two trials [26], [28], ≥6mmol/l in one trial [27], as significant hyperkalemia requiring treatment in one trial [29], and leading to discontinuation of study medication in another trial [24]. The definition of hyperkalemia was not reported in 3 trials [25], [30], [31].

Patients treated with combination therapy had a higher risk of worsening renal function than patients treated with ACE inhibitor therapy alone (RR 1.91, 95%CI 1.40–2.60, p-value for heterogeneity = 0.12; I^2^ = 46% [95%CI 0–80%]), symptomatic hypotension (RR 1.57, 95%CI 1.44–1.71, p-value for heterogeneity = 0.99; I^2^ = 0%[95%CI 0–79%]) and an increased risk of developing hyperkalemia (RR 1.95, 95%CI 0.85–4.48, p-value for heterogeneity = 0.007; I^2^ = 75%[95%CI 31–91%]). Trial medications were more often permanently discontinued in patients treated with combination therapy than in patients treated with ACE inhibitor therapy alone (RR 1.21, 95%CI 1.07–1.37, p-value for heterogeneity = 0.14; I^2^ = 38% [95%CI 0–74%]). There were no differences in the occurrence of cough, angioedema or rash between the two groups.

### Sensitivity Analyses

There were no qualitative differences in summary estimates for any outcomes of trials with and without concealed treatment allocation, double-blind design or blinded outcome assessment. Similarly, there were no qualitative changes in summary estimates for all outcomes when repeating the analyses after exclusion of those 2 trials that only included patients acute MIs [24], [25]. In this sensitivity analysis, however, there was no longer heterogeneity for hospital admissions due to congestive heart failure (RR 0.81; 95% CI 0.72–0.91, p-value for heterogeneity = 0.28, I^2^ = 23% [95%CI 0–88%]), but heterogeneity for the summary estimate of hospitalization for any reason did persist (RR 0.92; 95% CI 0.75–1.14, p-value for heterogeneity <0.001, I^2^ = 90% [95%CI 78–96%]).

There were no qualitative differences in summary estimates for any outcomes of trials that did respectively did not achieve ARB target doses although for admission for congestive heart failure heterogeneity for the summary estimates in trials achieving ARB target doses was reduced (RR 0.80; 95% CI 0.73–0.87, p-value for heterogeneity = 0.427, I^2^ = 0% [95%CI 0–90%]). In trials with ≥50% versus <50% of patients receiving beta-adrenergic antagonists, no difference in summary estimates were found in any outcomes. There were no qualitative differences in summary estimates for any outcomes when limiting the analyses to trials including more than 100 patients. Lack of individual patient data precluded us from conducting sensitivity analyses according to different etiologies (ischemic versus non-ischemic) or severity of heart failure (NYHA I and II versus NYHA III and IV).

## Discussion

In this meta-analysis, combination therapy of an ARB and an ACE inhibitor as compared to ACE inhibitor therapy alone did not reduce patient important cardiovascular outcomes such as overall mortality or non-fatal myocardial infarction in patients with left ventricular dysfunction or congestive heart failure. Combination therapy was associated with a reduction in patients' hospital admission for heart failure, but the risk for hospitalization for any reason was not affected. Combination therapy was associated with increased side effects such as worsening of renal function, hypotension and a tendency towards hyperkalemia in those trials where information was available.

Our study is based on trials retrieved from an extensive literature search to identify all relevant eligible trials comparing combination of ARB plus ACE inhibitor therapy to ACE inhibitor therapy alone in patients with congestive heart failure. In comparison to earlier published meta-analyses [15]–[18], our meta-analysis offers the advantage to simultaneously provide risk estimates on all patient relevant outcomes including hospital readmission rates for any reason which have not been reported in any of the previous meta-analyses.

Although formal testing did not indicate any publication bias, such bias cannot definitely be ruled out due to the small number of trials included and the low power of any test to detect a publication bias. Although only 3 of 8 included studies reported blinded outcome assessment, the quality of the included trials was generally good. In addition, the results of our analyses proved to be robust across various sensitivity analyses. Our analysis has several limitations. Only four trials aimed at reaching recommended full dose ARB therapy in the combination group [27]–[30]. Furthermore, in only 4 trials [26], [28], [30], [31] ≥80% of patients in the combination group reached the individual trials' target dose of the recommended ARB. It could therefore be argued that our results may underestimate the benefit of full dose combination therapy. However, in sensitivity analyses there was no qualitative difference in any of the outcomes analyzed when trials aiming at reaching recommended full dose ARB therapy were compared to trials where ≥80% of patients in the combination group reached the target dose of the recommended ARB. Although our sensitivity analysis may have lacked the power to demonstrate a difference in outcomes, it seems unlikely that higher doses of ARBs would lead to a greater benefit. In contrary, the fact that it proved difficult to reach recommended target dose of ARBs in individual trials most likely reflects the higher incidence of adverse effects associated with combination therapy. Any increase in the dose of ARBs in combination therapy may therefore lead to an even higher incidence of adverse effects without further benefit. In addition, only four trials [24], [26], [28], [31] reported to have aimed at using a fully recommended dose of an ACE inhibitor in the standard therapy group. Thus, the results of our meta-analysis may therefore rather over- than underestimate the beneficial effect of combination therapy.

The use of concomitant therapy with beta-adrenergic antagonists, a class of drugs with proven efficacy in the treatment of heart failure [34], [35], varied widely across trials, but was far from optimal. In only three trials [24], [28], [29] more than 50% of patients were taking beta-adrenergic antagonists in addition to ACE inhibitors and in only one small trial >90% were taking beta-adrenergic antagonists [29]. There is therefore insufficient evidence to answer the question whether the addition of ARBs to standard therapy with ACE inhibitors and beta-adrenergic antagonists offers any benefit in patients with heart failure.

In the absence of any benefit of combination therapy on mortality, the found effect on hospitalization due to heart failure may be of particular importance in view of the high costs associated with recurrent hospitalizations. Data on the cost-effectiveness of combination therapy compared to ACE inhibitor therapy alone for the treatment of congestive heart failure are scarce. In an analysis of resource utilization and costs in the Charm-added trial [36], combination therapy led to either cost-savings or small additional annual cost, depending on the country assessed. However, quality of life information was not incorporated into this analysis. Thus, treatment decisions favouring either therapy cannot be based on solid evidence due to the lack of any cost-utility analysis incorporating quality of life data comparing combination to ACE inhibitor therapy alone in patients with heart failure.

Individual trials included only very few patients with heart failure NYHA IV. Whether combination therapy proves beneficial in these patients with poor prognosis on standard therapy needs to be addressed by an adequately powered trial with strict monitoring of potentially dangerous adverse effects in this particular subgroup of severely ill patients. Only few patients of included trials had concomitant treatment with spironolactone, probably due to the increased risk of hyperkalemia when combining ACE inhibitors, ARB's and spironolactone. In the Randomized Aldactone Evaluation Study (RALES) [37], blockade of aldosterone receptors by spironolactone, in addition to standard therapy, substantially reduced the risk of both morbidity and death among patients with severe heart failure. In the ONTARGET trial combination therapy reduced proteinuria to a greater extent than monotherapy, however, overall renal outcomes were worse with combination therapy [38]. It is well known that blockade of the renin-angiotensin-aldosterone system results in an initial decline in renal function but may still prove beneficial in preserving renal function in the long-term. However, the results of our analysis demonstrate that the observed decline in renal function associated with combination therapy is detrimental to patients' health. Two trials defined decrease in renal function as either a ≥50% increase of serum creatinine from baseline [26], [27], and another trial [24] as one of the following: death from renal failure, end-stage renal disease requiring chronic dialysis or renal transplantation, or an increase in serum creatinine concentration leading to temporary or permanent discontinuation of study medication. In all 3 trials worsening of renal function occurred more commonly in patients assigned to combination therapy.

Generally, patients in clinical trials are more strictly monitored and adverse effects from combination therapy could have been detected earlier and more appropriately managed than under “real world” conditions. This has been well described for congestive heart failure patients with hyperkalemia treated with aldactone [37], [39]. Therefore, the higher incidence of adverse effects associated with combination therapy in randomized controlled trials may be even higher in clinical practice questioning the safety of combination therapy even further.

This meta-analysis confirms the findings from the recently published ONTARGET trial where no reduction in overall mortality but an increase in potentially serious side effects were found in patients at high vascular risk or diabetes treated with combination therapy [40].

### Limitations

Although we tried to obtain patient level data from primary investigators in order to conduct time-to-event and subgroup analyses, our request was not granted by some of the primary investigators. The lack of individual patient and aggregated follow-up data collected at uniform time points precluded us from analysing time-to-event data and from reporting absolute risk reductions and number needed to treat or to harm. Similarly, we were not able to identify relevant subgroups which may still benefit from combination therapy and we were not able to calculate risk differences that would allow for a better comparison of beneficial and adverse effects from combination therapy.

Our results have implications for guiding the direction of future research. There is a need for randomised controlled trials evaluating the effect of adding ARBs to patients taking ACE inhibitor therapy and beta-adrenergic antagonists for the treatment of heart failure in order to see whether ARBs offer any additional benefit to current standard therapy for heart failure. Alternatively, individual patient data meta-analysis may allow the identification of subgroups which potentially benefit more from combination therapy such as younger patients with preserved renal function ant thus at lower risk to experience worsening renal function or hyperkalemia.

### Conclusions

Combination therapy with ARBs and ACE inhibitors does not reduce mortality in patients with heart failure when compared to ACE inhibitor therapy alone. Although combination therapy does reduce hospitalizations for heart failure, it is associated with more adverse events and does not reduce all-cause hospitalization. Thus, based on current evidence, combination therapy with ARBs and ACE inhibitors may be reserved for patients who remain symptomatic on therapy with ACE inhibitors under strict monitoring for any signs of worsening renal function and/or symptomatic hypotension.
